# Surveillance of Nigerian children suggests that varicella may be a risk factor for acquisition of monkeypox

**DOI:** 10.3389/fpubh.2023.1140956

**Published:** 2023-02-09

**Authors:** Charles Grose

**Affiliations:** Division of Infectious Diseases, Virology Laboratory, Department of Pediatrics, University of Iowa, Iowa City, IA, United States

**Keywords:** varicella-zoster virus, chickenpox, mpox, smallpox, poxvirus

## Introduction

Both varicella and monkeypox are childhood viral diseases, each with its own distinctive exanthem. An intersection between the epidemiology profiles of varicella and monkeypox was not reported in a recent state-of-the-art review on monkeypox in children (1). Yet, Stephen et al. (2) have described several cases of coinfection with monkeypox and varicella during outbreaks of fever with rash in children and adults living in Northeastern Nigeria. In their Discussion, the authors speculate about the sequence of events during a coinfection with monkeypox and varicella. Therein lies the conundrum, namely, which infection came first in the child. In this Opinion, I side with the proposition that varicella came first. This Opinion is based in part on pathogenesis data collected by my research group decades ago on the common childhood illness varicella. Because of a recent recommendation by the World Health Organization to substitute the word monkeypox with the word mpox, mpox will be used below in this article (1).

## Patient population in Nigeria

Chickenpox is known to occur at a later age in children living in tropical countries, when compared with countries in temperate zones (3, 4). In one study in Nigerian school children, around 50% of children aged 4–6 years were seropositive for varicella antibody; this percentage rose to 70% by ages 13–15 years (5). The authors in the current study enrolled 33 patients with fever and rashes, but they eliminated 9 patients because their diagnoses could not be confirmed (2). Of the remaining 24 patients, 18 were children (up to age 19 years) and 6 were adults. The authors diagnosed 9 patients with a coinfection of varicella and mpox; of the 9 people, 5 were children. In addition, they found 2 patients with only mpox and 13 patients with only varicella. All diagnoses were confirmed by polymerase-chain reaction testing of skin lesions. Since the primary infection varicella (chickenpox) is caused by a herpesvirus (human herpesvirus 3; varicella-zoster virus; VZV) and mpox is caused by a poxvirus, there is no concern for any cross-reactivity in the identification of the correct virus.

Of special interest is the clustering of the 33 cases. There were two household clusters and one cluster of the older patients that occurred within prison. Again, of interest, one of the household clusters consisted of patients who were found to have only varicella. This analysis strongly suggests that there was an ongoing outbreak of varicella in the community at the time that mpox was also being detected.

## Pathogenesis of varicella (chickenpox)

What is not mentioned by the authors is the similarity in pathogenesis between varicella and poxviruses. The most thorough description of varicella pathogenesis was based on the pathogenesis of a poxvirus, namely mousepox (also called ectromelia) (6). Mousepox belongs to the same genus as mpox and they are closely related. The pathogenesis of mousepox was first delineated by the Australian virologist Fenner (7). In his oft-cited animal studies, he observed two viremic phases (8). In the first phase, the mouse acquires the infection through the nasal/oral route, after which there is viral replication in the head and neck. Then there is a primary viremia that seeds the major organs. After another cycle of replication in the major organs, there is a second viremia that leads to the exanthem of mousepox. In the model for varicella pathogenesis, a similar sequence of events was postulated, again with viremias followed by an exanthema (9).

In addition to an initial infection in the nose and throat, mousepox is often acquired *via* fomites that allow virus to enter through small breaks in the skin. Several outbreaks that have occurred in a laboratory animal facility (vivarium) have been blamed on contaminated bedding in the mouse cages, with entrance of virus *via* abrasions on the feet of mice (10, 11). Presumably a similar situation can occur in the houses of some of the mpox patients mentioned in the report from Nigeria, since rodents were observed in 86% of the houses (12). Rodents are considered to be the main reservoir of mpox in Africa (13, 14). If a chickenpox outbreak were occurring in the village, the chickenpox skin lesions over the entire body of a child would provide multiple portals for mpox virus on fomites to enter and infect a child. The minimum mpox inoculum required to infect a child could also be lower when mpox entered *via* a chickenpox skin lesion. Certainly, it seems that the minimum infectious mpox dose would be less *via* fomites entering a chickenpox skin lesion that *via* fomites inhaled in the nose and throat.

Further, the fomites with the crusts of mpox skin lesions within a household probably contain viable virus for months, based on the variola data (15). In contrast, VZV is a much more fragile virus (16). VZV is not known to survive for prolonged periods on inanimate objects in the environment. VZV is not known to infect rodents (17). The preponderance of evidence indicates that VZV is spread during close contact over a few hours in a room *via* the respiratory tract from a child with varicella to another child who has never had varicella (9). Otherwise stated, it is unlikely that a child with an early monkeypox rash would acquire varicella virus by entry of VZV-contaminated fomites through the mpox skin lesions.

## Incubation periods

**Figure 1:**
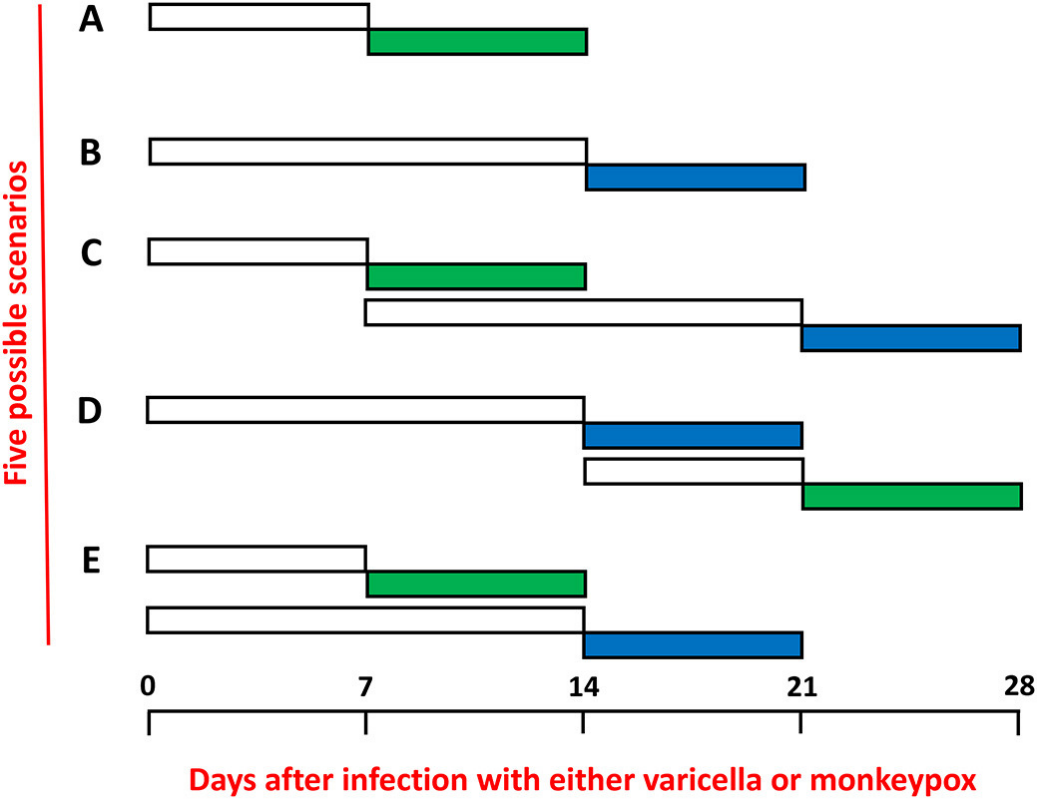
Incubation periods and exanthems of mpox and varicella. The incubation periods are shown in non-colored rectangles and the periods of the exanthems are shown in colored rectangles: mpox (green) and varicella (blue). The time periods are expressed as numbers of days separated into weeks. **(A)** Mpox only. **(B)** Varicella (chickenpox) only. **(C)** Sequential infections with mpox first and varicella second. **(D)** Sequential infections with varicella first and mpox second. **(E)** Simultaneous infections beginning on the same day with both mpox and varicella.

Further the incubation period of mpox is shorter than the incubation period of varicella. Recent studies have suggested an average time period of 7–8 days for mpox (Figure 1, row A), while many older studies have defined the incubation for varicella as 14–15 days (Figure 1, row B) (9, 18, 19). Therefore, a child with early varicella could acquire mpox and still be in the acute illness when mpox erupted 7 days later (Figure 1, row D). However, if a child with mpox acquired varicella, there should be a lag period of 14 days before the varicella exanthem appeared (Figure 1, row C). Altogether therefore under the mpox/varicella scenario, the duration of the rash would be prolonged by a week or more. The least likely scenario is that the children with coinfection contracted both viruses on the same day (Figure 1, row E), Under the latter scenario, each child would need to be exposed on the same day to a source of varicella (another infected child) and a source of mpox (another infected child or a house contaminated with the poxvirus on fomites).

## Epidemiology of herpes zoster

Herpes zoster (shingles) is the name of the disease that represents a reactivation of the same VZV strain that infected a child during the disease chickenpox (varicella) (17). Following varicella, the virus remains latent in the dorsal root ganglia, until it emerges decades later and travels along a sensory nerve to a dermatome, where a rash appears. Generally, the rash is restricted to one or two dermatomes on one side of the body, allowing an easy diagnosis simply by observation. Occasionally, the papulovesicular rash spreads over the body; Under this circumstance, the rash can be confused with varicella, the primary infection. However, there is no evidence in multi-year surveys of herpes zoster in children that outbreaks of this disease can occur in otherwise healthy children (20, 21). Although few epidemiology studies of varicella and herpes zoster have been published in African children, there is no reason to expect more frequent herpes zoster in African children than observed in children elsewhere in the world (22). Since there is no evidence that herpes zoster could occur in a cluster in an African village, there is no precedent for 9 children with coinfection to have had simultaneous herpes zoster and mpox in Nigeria. By this reasoning, the most likely explanation is that the children with rashes diagnosed by VZV testing in this study had varicella and did not have herpes zoster.

## Discussion

For all the above reasons, the simplest explanation for the Nigerian outbreaks is that varicella occurred first, followed rapidly by a second infection with mpox. The clustering of cases suggests that there were outbreaks of varicella in the villages where mpox was found (2). Since there were far more cases of varicella than mpox, the data further suggest that a subgroup of children with varicella contracted mpox. If rodents infected with the poxvirus were living within a house where several children had varicella, the poxvirus could enter through the varicella skin lesions and cause a coinfection. Based on this hypothesis, varicella infection is a risk factor for acquisition of mpox.

In their Discussion, the authors propose that it may be worthwhile for the varicella vaccine to be added to the routine vaccination schedule for children in Nigeria (2). The rationale was to eliminate the confusion over diagnosis of varicella vs. mpox in children. However, if varicella really is a risk factor for acquisition of mpox, another option would be to conduct an interventional trial in which one cohort of children is given varicella vaccination over a few years and a second cohort is not given vaccination, in order to determine whether mpox declines more in vaccinated children than in a comparable cohort that is not vaccinated. In other words, varicella vaccination would lead to fewer cases of mpox in Nigerian children. This positive result would greatly expand the rationale for universal varicella vaccination of Nigerian children.

## Author contributions

CG reviewed the literature, then wrote the original and revised manuscript, and has read and accepts the revised version of the manuscript.
